# Sclerotherapy for the intraoral ranula with bleomycin: technical considerations and preliminary experience

**DOI:** 10.1186/s12903-024-04633-8

**Published:** 2024-07-24

**Authors:** Hong-Yu Zhang, Li-Wen Su, Huan Sun, Chao-Chen Rui, Yang Wu

**Affiliations:** 1 State Key Laboratory of Oral & Maxillofacial Reconstruction and Regeneration, Key Laboratory of Oral Biomedicine Ministry of Education, Hubei Key Laboratory of Stomatology, School & Hospital of Stomatology, Wuhan University, Wuhan, China; 2 Department of General Dentistry, School & Hospital of Stomatology, Wuhan University, Wuhan, China; 3 Department of Oral and Maxillofacial Surgery, School & Hospital of Stomatology, Wuhan University, Wuhan, China

**Keywords:** Sublingual Gland, Ranula, Bleomycin, Sclerotherapy

## Abstract

Ranula is a mucous cyst that occurs in the sublingual gland (SLG) in the floor of the mouth. It can be classified into two types based on origins: One is the the lesser sublingual gland (LSLG) in the anterior segment and the Rivini duct, which is connected to it, and the other is the greater sublingual gland (GSLG) in the posterior segment. Because of the anatomical characteristics, surgical resection of the cysts carries the risk of damaging adjacent tissues and has a high recurrence rate. Intralesional injection of sclerotherapy may be a better alternative treatment. We summarized 65 cases of ranula treated with intralesional injections of bleomycin(BML). According to the origin of the ranula, 60 cases were from the LSLG and the Rivini duct, and 5 cases were from the GSLG. The results showed that 60 cases of ranula from LSLG and Rivini ducts were 100% cured during the follow-up period. The median number of injections for all patients was 1.16. All 5 cases of ranula from the GSLG did not wholly recover. This study confirmed that BLM intralesional injection is a safe and effective treatment modality for cysts from LSLG or the ducts of Rivini rather than GSLG. Therefore, before treatment, it is necessary to determine the type and origin of the cyst by characterizing its morphology to ensure the effectiveness of the treatment.

## Introduction

Mucous cysts are the most common benign masses in the oral cavity. When located in the oral floor, they're called ranula, originating from salivary glands in the oral floor [1]. The general classification of ranula is usually divided into two forms: a simple (or intraoral) ranula and a plunging (or cervical) ranula. The intraoral type is confined to the upper part of the mandibular hyoid muscle. In contrast, Type I the plunging type extends along the fascial plane down to the cervical, submandibular, or sub-chin space [2]. This is a classification by the orientation of the bulge of the cyst. If the classification is based on the anatomy of the sublingual gland (GSL) and the origin of the ranula, there are two sources of ranula: One is the greater sublingual gland (GSLG) in the posterior segment, and the other is the lesser sublingual gland (LSLG) in the anterior segment and the Rivini duct attached to it. They can also be categorized into two groups based on clinical presentation. Type I is the mucous cyst of GSLG, which is usually larger and presents as a dark blue, rounded mass. It may present as intraoral or plunging [3, 4]. Type II is the cysts from the LSLG or the Rivini duct, which is clinically small, rarely exceeding 15 mm, localized to the intraoral without plunging [5, 6].

Ranula are relatively uncommon, and its proportion in oral mucoceles is about 9.25% [7]. Usually, surgical removal of the cyst and the SLG is the accepted protocol for Type I of the ranula. In previous studies, most articles did not strictly differentiate between the diagnosis and treatment of the different sources of ranula. As a result, some Type II ranula also underwent concomitant resection of the SLG, which has the problem of over-surgery. However, for the second type of ranula, complete removal of the small glands alone may be complicated by the complexity of the anatomy of the floor area of the oral cavity. If the removal is incomplete, postoperative recurrence is susceptible. As a result, researchers have been widely interested in exploring a safe and effective treatment alternative to surgical resection. Sclerotherapy refers to using chemical injections to stimulate the formation of fibrous connective tissue locally in the body, which causes the lesion to sclerotize and atrophy, thereby eliminating the lesion or treating the disease [8].

Bleomycin(BLM) sclerotherapy has been widely used to treat various cystic diseases. Its mechanism of sclerogenic action can be hypothesized to be the destruction of endothelial cells through a nonspecific inflammatory response in the early stage and the collapse and atrophy of the cyst by tissue fibrosis in the late stage. Based on the sclerosing mechanism of BLM, we hypothesized that it is a safe and effective treatment for intraoral ranula and tried to apply it in such cases. This study aims to observe the efficacy of BLM local injection in treating intraoral ranula.

## Materials and methods

From September 2020 to September 2023, a total of 65 patients with ranula were treated with intralesional injection of BLM at the Department of Oral and Maxillofacial Surgery, Hospital of Stomatology, Wuhan University, and followed up for more than 6 months after the final treatment. There were 29 males and 36 females, aged 5-74 years, with a median age of 22.33. Informed consent was obtained in all cases (Table 1). Approval was granted by the Wuhan University Medical Ethics Committee. BLM solution (concentration, 2.5 mg/ml) was made by dissolving 15 mg of BLM in 5 ml of lidocaine hydrochloride and 1 ml of dexamethasone, and the appropriate volume of solution was taken according to the size of the cyst.

**Table 1:** Clinical characteristics of the patients

**Characteristics**	**Number of patients (%)**
Age (years)	
Maximum age	74
Minimum age	5
Average age	22.33
**Sex**	
Male:	29(44.61%)
Female:	36(55.38%)
**Size of the lesion (mm, in diameter)**	
From the lesser sublingual gland & Rivini ducts cysts	
Maximum size	7
Minimum size	20
Mean ± SD	10.54 ± 3.48
From the greater sublingual gland	
Maximum size	17
Minimum size	22
Mean ± SD	19.66 ± 1.35

**Fig. 1:**
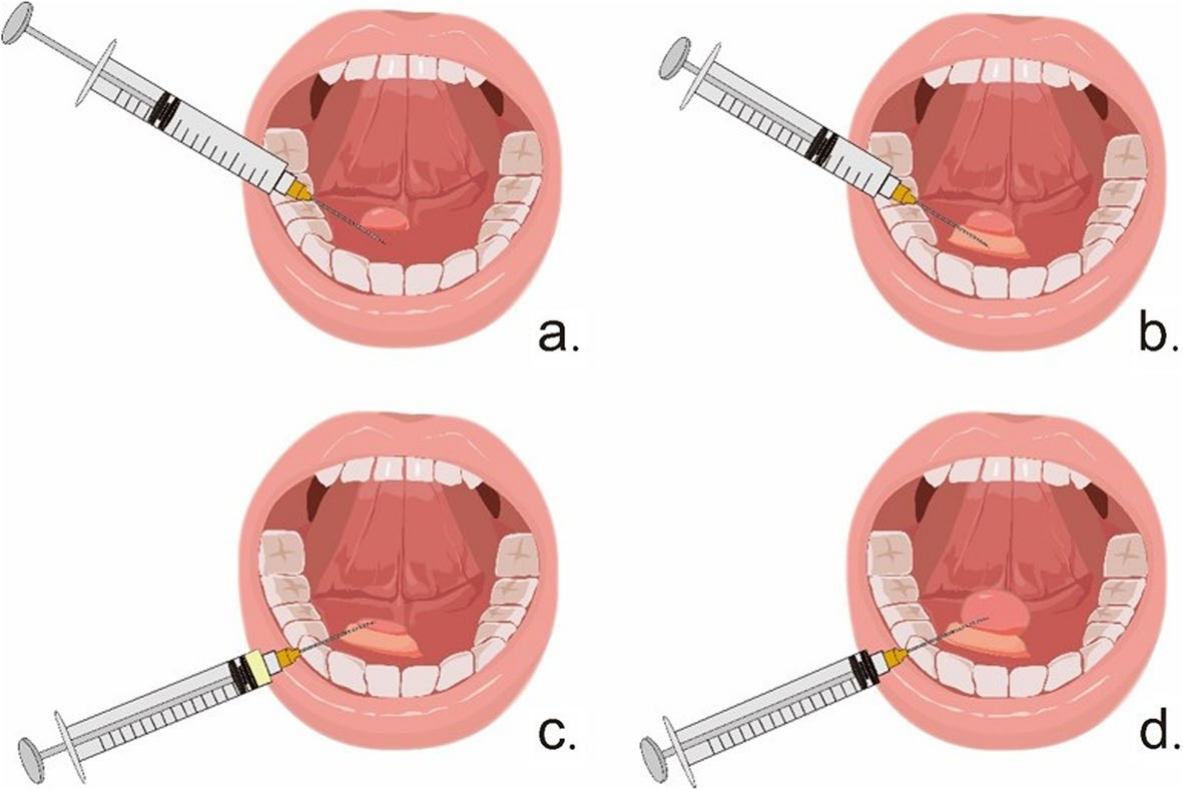
Schematic diagram of injection therapy. **a** The needle is inserted into the healthy mucosal tissue surrounding the cyst at an angle of approximately 75 degrees to the mucosa, and the injection site is directed to the base of the cyst. **b** One-half of the total dose of BLM solution (2 mg/ml) is injected, and the base of the cyst swells. **c** The needle is slightly backed up and inserted into the cystic cavity, an empty tube syringe is replaced to aspirate the mucus, and the ranual shrivels up. **d** Replace back to the syringe with the BLM solution and inject the remaining BLM solution into the vesicle lumen until the lumen is full and unbroken

The injection process is divided into two steps. First, the injection needle was inserted into the mucosal tissue 5 mm from the peripheral edge of the cyst at an angle of approximately 75 degrees to the mucosa, and the injection site was directed to the base of the cyst. Half of the total dose is injected, and a localized elevation of the cyst site is seen. The needle is then slightly backed up and inserted into the cystic cavity, sometimes accompanied by a puncture drop. For cysts larger than 10 mm in diameter, it is necessary to switch to an empty syringe to aspirate as much cystic fluid tissue as possible. Cysts less than 10 mm in diameter are usually unable to aspirate the cystic fluid, so the remaining BLM solution can be injected into the cyst directly. The endpoint of the injection is based on the lesion slightly expanded and the surface mucosa of superficial lesions turning pale (Fig. 1). All patients returned visited every 3 weeks after the first injection until 12 weeks and then every 3 months. If necessary, the injection was repeated after 3 weeks. All procedures were performed in an outpatient clinic without hospitalization.

The criteria for evaluating efficacy are as follows: Complete regression: complete regression of the cyst, with no obvious abnormality of the mucous membrane and no scarring or recurrence observed during follow-up. General improvement: the cyst shrinks into a hard or immobile painless nodule with whitening of the mucosa; no recurrence observed during follow-up. Ineffective: no significant change, recurrence, or regeneration of the mucus cyst. Treatment is considered successful when complete resolution or general improvement is achieved.

## Results

All 65 cases were first-time offenders. After 1-3 intralesional injections, 39 cases achieved complete regression, 21 achieved general improvement, and 5 were ineffective. These 5 ineffective cases were diagnosed as the greater sublingual gland origin ranual prior to treatment and did not show any improvement after the first injection, thus the decision was made to refer them for surgical treatment after discussion with the patients. Among the successfully treated 60 cases, 52 underwent one injection, 6 underwent two injections, and 2 underwent three injections. The total number of injections in successfully treated cases was 70, with an average of 1.16 injections per patient (Table 2). In this series, all patients recovered entirely without recurrence except for 5 cases of inappropriate indication selection.

**Table 2:** Outcomes of the patients

**Outcomes**	**Number of patients (%)**
**Treatment outcome**	
Complete resolution	39 (65.00%)
General improvement	21 (35.00%)
Ineffective	5
**Treatment episodes** (Except for five ineffective cases)	
1	52 (86.67%)
2	6 (10.00%)
3	2 (3.33%)
**Cure rate**	
LSLG & Rivini ducts ranula	100%
GSLG ranula	0%
Total	92.31%

**Fig. 2:**
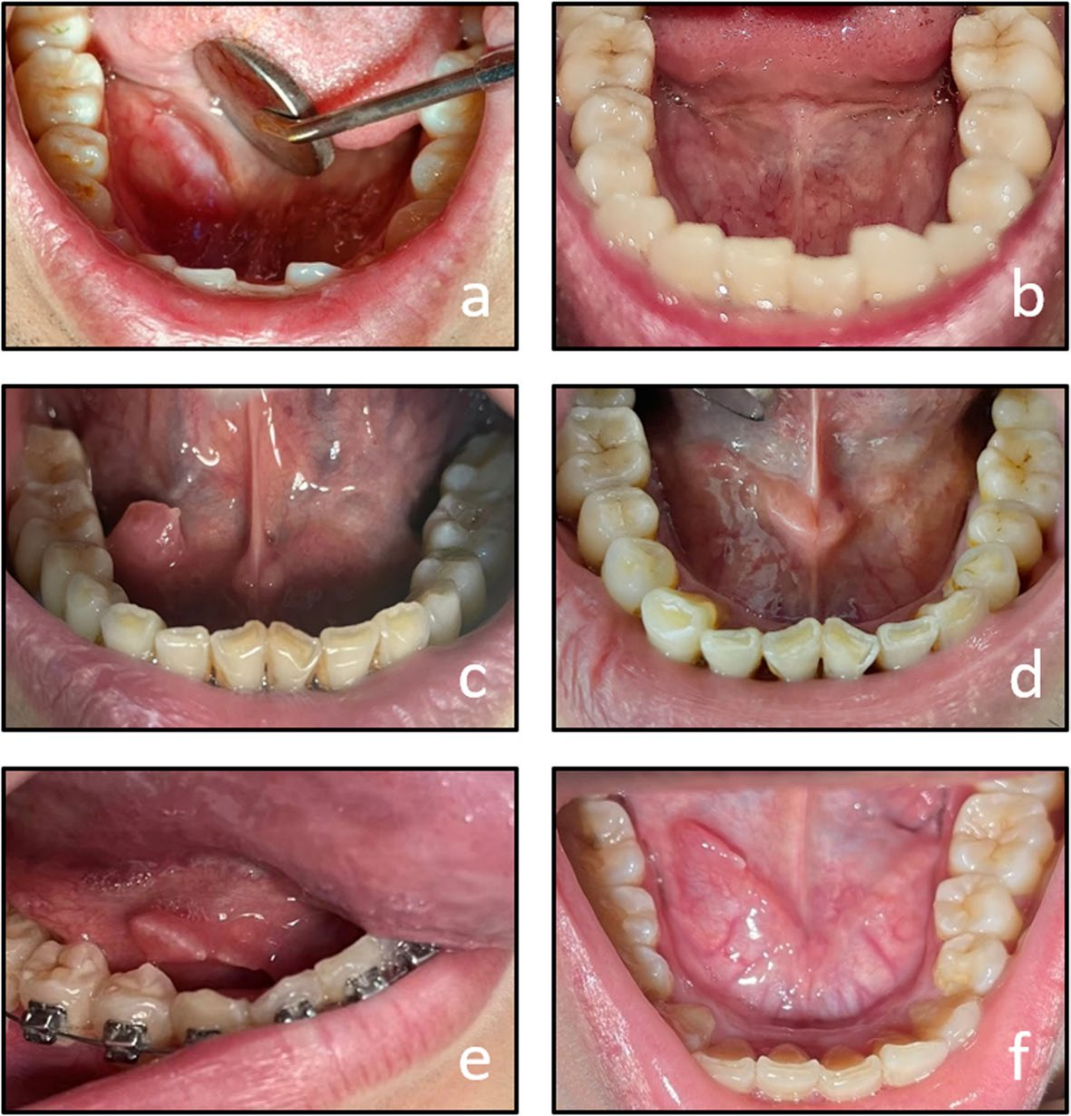
Before and after BLM sclerotherapy. **a** & **b** a case treated with a single injection **c** & **d** a case treated with twice injections **e** & **f **a case treated with three times injections

Patient’s reaction after injection can be broadly divided into three stages. Stage 1: The filled cyst slowly shrank to its size before injection. Stage 2: The cyst continued to shrink, accompanied by local edema and pain. In some patients, the cysts are separated into several small blisters. Stage 3: The size of the cyst significantly decreased until it disappeared or stabilized into a fixed-sized nodule, with no apparent discomfort around the injection site. In some patients, mild or moderate swelling and pain at the injection site subsided within 1-3 days after injection, with an average duration of 3.27 days. Ulcers appeared in 7 cases on the second day after injection and subsided within 4-9 days, with an average ulcer healing time of 5.5 days. There were no other complications associated with the injection, including localized hypertrophy and pulmonary fibrosis (Fig. 2).

## Discussion

**Fig. 3:**
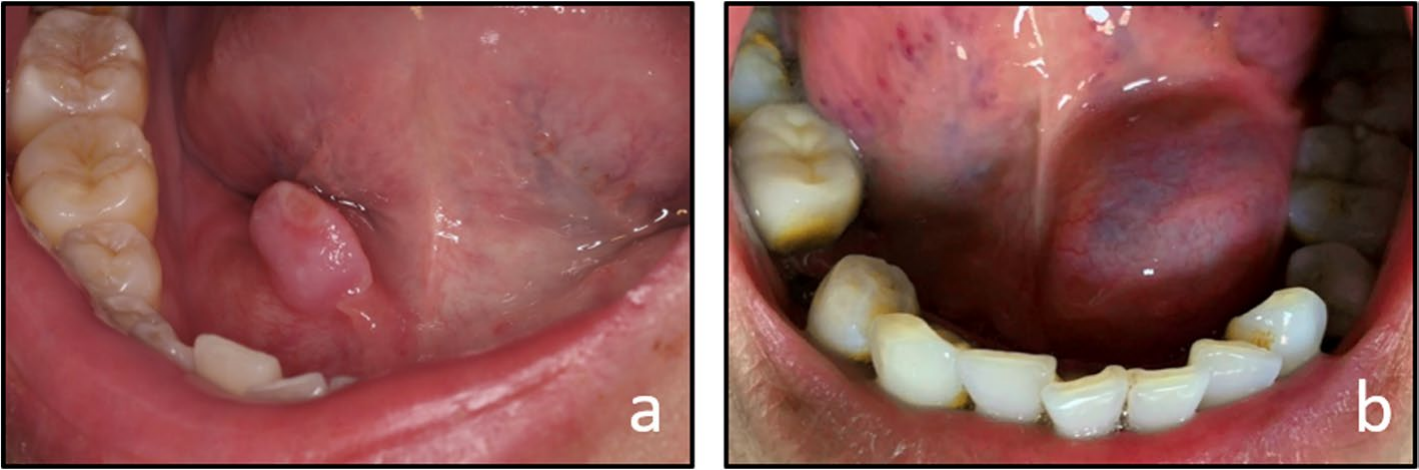
Comparison of the ducts of Rivini cyst and sublingual gland cyst. **a** Ranula from the ducts of Rivini is shallow, usually no more than 1.5 cm in diameter, red to clear in color, sometimes with a white surface due to scarring **b** Ranula from GSLG is large and deep, red and blue in color

Oral mucous cysts are common oral mucosal lesions. When the cyst occurs in the floor of the oral, they are called ranula because the swelling resembles a frog's air sac [1]. The general classification of ranula is usually divided into two forms: a simple (or intraoral) ranula and a plunging (or cervical) ranula, which is categorized by the orientation of the cyst's bulge. Ranula is derived from the SLG, so the classification of ranula is closely related to the anatomy of the SLG. In this article, we classify the cysts according to their origin. The SLG consists of a constant lesser sublingual gland (LSLG) and a greater sublingual gland (GSLG). The GSLG is located behind the LSLG in the paralinguinal space. This structure is not constantly present. The GSLG is a distinct structure that drains via a single duct and either connects to Wharton's duct or opens independently. The LSLG consists of several small glands, each of which is traversed by a Rivini duct that opens independently in the floor of the oral [9–11]. We have combined the anatomy of the SLG with the clinical presentation of the different types of ranula to categorize them into two forms. Type I is a mucous cyst originating from the GSLG [3, 4], the most typical ranula. They are usually larger and deeper, characterized by soft and painless subcutaneous fluid accumulation. Due to tissue cyanosis and vascular congestion, its color varies from dark blue to normal pink. They can present as intraoral or plunging types. Type II is a cyst arising from the SLG or from the Rivini duct. They are usually small to medium-sized, rarely larger than 15 mm, and are always superficial, appearing red to clear in color and sometimes whitening due to thickening of the ruptured surface, which is generally confined to intraoral (Fig. 3) [12] .

Currently, the mainstay of treatment for ranula remains surgical removal of the sublingual gland and cyst, which is considered the most reliable method because it rarely leads to recurrence [13, 14], despite its high invasiveness [15, 2]. Subsequently, surgical procedures such as marsupialization have been introduced by surgeons to minimize trauma in the treatment of sublingual gland cysts [16, 17]. Later, the cryosurgical technique, carbon dioxide laser radiation, and other therapies have been used to treat sublingual gland cysts [18–20]. Baurmash suggests that unconditional removal of the sublingual gland should not be the standard treatment for all ranula, as some ranula originating from the sublingual gland have a low likelihood of recurrence, and even some cases may not originate from the sublingual gland [21]. Treatment of such mucous cysts should involve standard marsupialization followed by evacuating the liquid or completely removing the accessory glandular tissue. In 2008, McGurk et al. [22] proposed a method of treating ranula by partial excision of the SLG, based on the principle that ranula is supplied by discrete units of the sublingual gland attached to the gland. Therefore, it can be treated by removing only the portion of the sublingual gland attached to the ranula, which preserves a large portion of the sublingual gland. It was hypothesized that a cure for ranula could be achieved if the origin of the cystic fluid production could be localized and the localized extravasated glands eliminated. However, unlike mucous cysts of the buccolabial region, the complex anatomy and deeper location of the oral floor make surgical procedures inconvenient [23]. Moreover, removal of the oral floor minor salivary gland cysts alone has also been shown to be unreliable [14]. Due to the complex anatomy of the oral floor region, accurate localization and excision of the glands surrounding the cyst is difficult and entails incisional trauma. Moreover, incomplete removal of the extravasated mucus gland or intraoperative rupture of other small follicles can potentially lead to postoperative recurrence. Therefore, there is an urgent need to introduce safer and more effective treatment modalities.

Sclerotherapy has previously been introduced to treat mucous gland cysts [24, 25]. In 2003, Fukase first used OK-432 for the treatment of sublingual gland cysts based on their experience in treating lymphangiectasia [24]. In 2017, Cai et al. [26] conducted a study involving 40 patients with the mucoceles of the anterior lingual salivary glands treated with pingyangmycin as a sclerosing agent. All cases were cured without recurrence after more than 16 months of follow-up. In 2018, Liu et al.^27^ reported polidocanol sclerotherapy in the mucocele of the minor salivary gland in 112 patients, with an overall cure rate of 91.07%. In 2021, Huang et al.^28^ used promethazine hydrochloride injections to treat mucous cysts of the minor salivary glands, with a cure rate of 96.8%.

Mucous cysts are similar to lymphangiomas because they are both thin-walled cystic diseases. Therefore, we believe that intracapsular injection of BLM may be as effective in treating cysts as in treating lymphangiomas. In this study, we extended the application of BLM to ranula. BLM analogs are a family of glycopeptides isolated from cultures of Streptomyces verticillus by Hamao Umezawa in 1962 [29] BLM analog sclerotherapy has been successfully used for decades in the treatment of lymphatic malformations, hemangiomas, angiomata, cystic craniopharyngiomas, and bronchial cysts [30]. The mechanism of sclerotherapy of cysts with BLM is hypothesized to be inflammatory exudation of the cyst wall in the early stages, followed by collapse and atrophy of the lumen due to tissue fibrosis in the late stages [31].

In our clinical practice of BLM sclerotherapy injections of these ranula from the LSLG or Rivini ducts, it has been found that the cysts slowly decrease in size due to the absorption of the drug for a period of time after the end of the injection. Then, in the early stages of sclerotherapy, there is a sensation of swelling and pain, and there is a recolonization of the cystic fluid, which sometimes appears to be separated into several blisters. In the later stage of sclerotherapy, the size of the cysts decreases significantly and disappears or stabilizes to a fixed size. The timing of each stage was not consistent between cases, and it is hypothesized that the size of the cysts and whether or not they ruptured are factors in their influence. For the five cases of GSLG, there was no reduction of the cysts after intra-lesion injection of BLM. The possible causes are as follows. The GSLG is located further back and deeper in the floor of the mouth. The cystic cavity is usually larger and contains more cystic fluid. As the cystic fluid is withdrawn from the cyst, the gland rapidly produces new mucus, which results in dilution of the drug, preventing BLM from remaining in the cystic lumen at a high concentration and acting on the surrounding glands that have become diseased, resulting in insufficient fibrosis of the gland with mucus extravasation. Besides, the mucus that initiated the cyst is produced from a remote location. Mucus extravasated glands cannot be adequately infiltrated by accurate localization. When BLM is injected into the cyst, the glands around the injection point will be inducing fibrosis, but the distant initiation point did not come into contact with the drug. This will result in the inability to eliminate the generation of mucus, making treatment ineffective. From this, we concluded that injection therapy did not yield the desired results for deeply located ranula and therefore did not attempt treatment for plunging ranula. A 2014 study by Manner et al.^32^ had similar results. Among the 18 cases they included, complete response was achieved in only 22% of cases after a course of at least three aspirations of cyst fluid, doxycycline lavage followed by BLM injection. In fact, the criteria for inclusion of cases in the article were patients with a plunging or intraoral ranula. However, the clinical characteristics of the cysts in the cases that were cured were not additionally documented in the article, so it is not possible to determine whether this technique is uniquely curative for a particular type of ranula. Based on the available results, it can only be stated that sclerotherapy with bleomycin is indeed not suitable for all ranula. However, at the same time, the injection method, sclerotherapy volume, and other undocumented factors may affect the cure rate of this treatment modality, so there is still further discussion about the cure rate mentioned in this literature. In this study, it was confirmed that BLM sclerotherapy does have a unique curative effect on a specific type of ranula by categorizing the anatomical source of the ranula, but it is not as effective for types of ranula other than this. Based on the experimental results in this study, direct complete excision of the sublingual gland or other treatments with higher success rates are more recommended for ranula from GSLG than repeated injections and recurrences.

Regarding complications, BLM produces direct chemical irritation injury in addition to its cytotoxic effects. The most common complication was localized pain and ulcers at the injection site. According to follow-up statistics after injections, the average duration of pain was 3.27 days, and ulcer healing time was 5.5 days. We recommend using surface anesthetics to alleviate local pain. It is reported that there is a risk of pulmonary fibrosis when the cumulative dosage of BLM exceeds 450mg. In this study, the risk of pulmonary fibrosis and local excessive atrophy was extremely low due to the use of a small dosage and a three-week injection interval [33].

To maximize the efficacy and improve the safety of BLM sclerotherapy, we made a solvent of 15 mg BLM powder with 5 ml of 2% lidocaine and 1 ml of dexamethasone before injection. Lidocaine can reduce pain after injection. Dexamethasone can prolong the preservation time of the drug in the lesion and enhance the effectiveness of the drug by constricting local blood vessels.

In our previous attempts, we injected directly into the cyst and found that when the cyst was small, it was more difficult to stab directly into the interior of the cyst. This method often resulted in puncturing the base of the cyst or rupture of the cyst cavity due to overfilling. However, the requirement for injection is to ensure sufficient BLM solution remains in contact with the entire lesion. So, we improved the injection method in this study. Firstly, BLM solution is injected into the base of cysts. Mild swelling of the basal tissue can block the BLM solution and delay absorption. Subsequently, insert the needle into the cyst from the bottom, extract the cystic fluid if possible, and then inject BLM to fill and bulge the cyst. It is essential to avoid the rupture of the cyst caused by excessive filling, which may lead to the outflow of BLM.

We found that when a patient reported a recent cyst rupture, the first injection usually failed to achieve satisfactory results. The reason may be that the rupture in the cyst allows the drug to outflow and prevents it from being in sufficient contact with the cyst surrounding for a sufficient period of time, affecting the treatment's efficacy. In this study, the cure rate of ranula of LSLG and the duct of Rivini origin was 86.67% for the first injection, and the final cure rate was 100% after 1-3 injections, so the injection of BLM under the appropriate method is very effective in treating superficial ranula.

## Conclusion

Bleomycin injection sclerotherapy is an efficient, safe, simple, cheaper, and minimally invasive treatment for the ranula from LSLG and Rivini ducts.

## Data Availability

All data generated or analysed during this study are included in this published article.
